# Odometry and Laser Scanner Fusion Based on a Discrete Extended Kalman Filter for Robotic Platooning Guidance

**DOI:** 10.3390/s110908339

**Published:** 2011-08-29

**Authors:** Felipe Espinosa, Carlos Santos, Marta Marrón-Romera, Daniel Pizarro, Fernando Valdés, Javier Dongil

**Affiliations:** Electronics Department, Higher Polytechnic School, University of Alcala, Alcalá de Henares 28805 Madrid, Spain; E-Mails: carlos.santos@depeca.uah.es (C.S.); marta@depeca.uah.es (M.M.-R); pizarro@depeca.uah.es (D.P.); fernando.valdes@depeca.uah.es (F.V.); javier.dongil@uah.es (J.D.)

**Keywords:** Kalman filter, sensor fusion, intelligent robots, data processing, robot control, laser application, dead reckoning, state estimation, multirobot system, robot sensing system

## Abstract

This paper describes a relative localization system used to achieve the navigation of a convoy of robotic units in indoor environments. This positioning system is carried out fusing two sensorial sources: (a) an odometric system and (b) a laser scanner together with artificial landmarks located on top of the units. The laser source allows one to compensate the cumulative error inherent to dead-reckoning; whereas the odometry source provides less pose uncertainty in short trajectories. A discrete Extended Kalman Filter, customized for this application, is used in order to accomplish this aim under real time constraints. Different experimental results with a convoy of Pioneer P3-DX units tracking non-linear trajectories are shown. The paper shows that a simple setup based on low cost laser range systems and robot built-in odometry sensors is able to give a high degree of robustness and accuracy to the relative localization problem of convoy units for indoor applications.

## 1. Introduction

In the past, mobile robot cooperation has been widely studied in multiple application scenarios. Multi-robot systems exhibit advantages with respect to single-robot systems, in terms of flexibility, adaptability, scalability and affordability. However, localization, communication and control challenges are more significant in cooperative robotics.

From the point of view of localization, two kinds of scenarios can be considered: the first one requires a global localization of each unit independently (e.g., swarm applications [1]). In the second one, only relative localization between robots is required (e.g., convoy applications [2], where only convoy leaders may need global localization).

The selection of the sensorial systems needed for robot localization is a crucial task that depends heavily on the application scenario (*i.e*., indoor or outdoor environments). Localization in outdoor scenarios can be easily performed by a combination of GPS systems and relative localization sensors (e.g., mid-range laser scanners, odometry, *etc*.). On the contrary, localization in indoor environments is a challenging and still unsolved problem in some aspects. Indoor GPS systems using a wide variety of sensor technologies (e.g., vision, ultrasound, infrared, *etc*.) are mostly in the research state. This paper deals with the problem of relative localization for cooperative guidance of robotic units in a convoy, considering non-linear trajectories in indoor environments.

Multi-sensory strategies are usually proposed to solve the relative localization problem, where odometry information (*i.e*., originally included in most of the robots and prone to add cumulative errors), is combined with other sensors, such as laser, ultrasound or vision. In general, the accuracy of these technologies is highly dependent on the sensor setup. Considering only sensors onboard the robots, the localization accuracy depends on several factors, such as cost, number of sensors, complexity and limitations of each technology. This paper proposes to include a sensor on top of each robot that is able to give position and orientation of the next robot unit in a robotic convoy. In this context laser rangefinder accuracy is higher than the one based on sonar (ultrasound) [3,4], using either natural shape of the robot or with artificial landmarks on it. On the other hand, computational vision is able to easily improve the laser accuracy using visual landmarks [5] at a relative low cost. However the cost of the setup and its complexity increases when it is necessary to make the system resistant to varying illumination conditions (e.g., active infrared landmarks) and to operate at high frame rates.

This paper shows that a simple setup based on low cost laser range systems and built-in robot odometry sensors is able to provide a high degree of robustness and accuracy to the relative localization problem of convoy units in indoor environments. Aside from the localization problem, the design of a control strategy for each individual unit in the convoy presents important challenges. In order to follow the leader’s trajectory it is not enough to guarantee global stability. A movement coordination plan is also needed between at least each pair of consecutive units [6]. This coordination involves exchanging continuously the motion state between convoy units, as explained in works such as [7–10]. To successfully achieve convoy navigation it is essential to have a highly reliable and exact positioning system providing the convoy leader with its global pose and each convoy unit with its relative localization to the preceding unit.

Among the published works regarding convoy guidance for indoor applications, the following should be mentioned: In [11] a sensorial system is designed for the high level navigation of a convoy for indoor construction site security and safety. The proposed on-board sensorial system in the robots (ultrasonic range modules, infrared distance measuring devices, colour camera, microphone and speaker) is complemented by wireless sensor networking devices. In [12] a vision system can recognize and relatively localize the follower robots using markers mounted on the leader unit. In [13] the indoor localization problem for convoy guidance is solved using a camera and colour signboard landmarks placed in the environment. A Kalman Filter and an Interacting Multiple Model method are applied to find the robots accurate positions and identify them by using the signboards. In [14,15] a demonstrator of a leader and four followers is described, where relative localization between convoy units is solved by means of a Sick LMS 200 laser rangefinder (LIDAR) and the robots themselves (Pioneer 2-DX) are used as landmarks. The Sick LIDAR sensor proposed had 0.25° of angular resolution, 15 mm of depth resolution and 10 m range. Although using an accurate sensor, the aforementioned work does not include odometry information and it thus relies only on the laser measurements.

**Figure 1.**
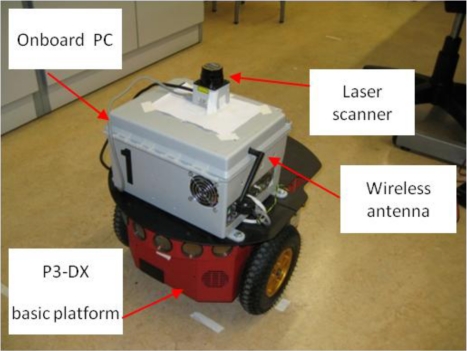
One of the P3-DX robotic platforms equipped with encoders and laser scanner for the platoon guidance demonstrator.

**Figure 2.**
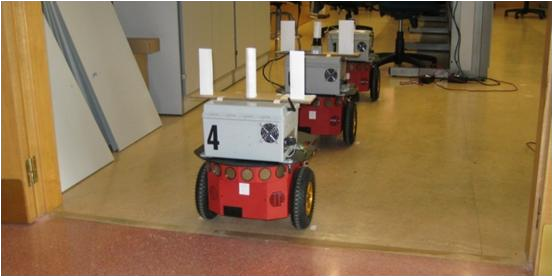
Convoy of three robotic units as the one shown in Figure 1. Landmarks related to the laser scanner system are onboarded on top of the robots.

In the light of the previous works the main contribution of this paper is to implement an innovative and low cost relative localization system for a convoy of robotics units in indoor transport scenarios. The LIDAR sensor proposed in this paper is a Hokuyo URG-04-LX [16,17]. Its performance is remarkable lower (0.36° of angular resolution, 40 mm of linear accuracy and 4 m range) than the previous mentioned Sick LIDAR, but the cost is about six times cheaper. To compensate its accuracy this paper proposes to combine laser measurements with odometry. This way, the algorithm is able to profit from the high resolution of odometry (1.2 mm of resolution) in short movements at the same time non-cumulative error is compensated in large trajectories using laser measurements. Besides, odometry sensors operate at high frequency (50 ms in the application described in this paper), which allows to maintain relative localization accurate whether a momentary blinding happens; that is not possible using only laser, camera or sonar devices. To summarize, data fusion makes it possible to combine the positioning data of the robot odometric system (with a low uncertainty but cumulative error) and an on-board laser scanner in the follower units (with non-cumulative errors). A solution based on the discrete Extended Kalman Filter (EKF) is proposed. Units used in the convoy demonstrator are based on P3-DX robots from MobileRobots [18,19], that have been adapted to the requirements of the proposed scenario with different electronic devices (see Figures 1 and 2); some of them were designed *ad-hoc* [20,21].

**Figure 3.**
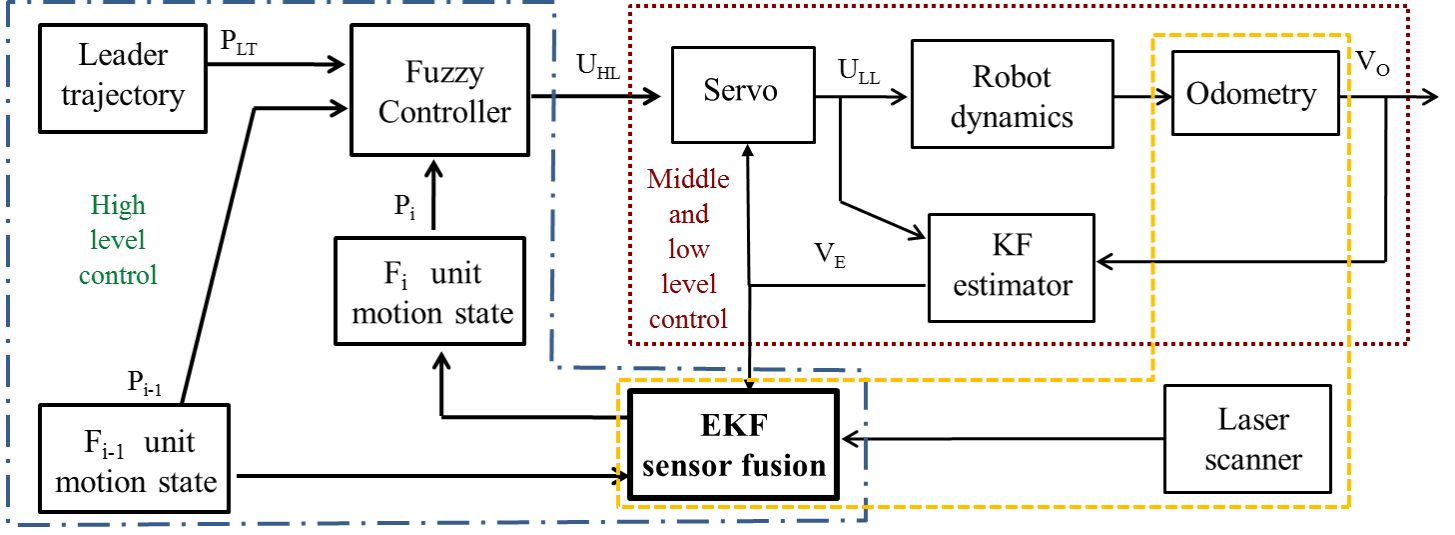
Organization of the control levels and their relation with the sensorial systems included in each follower unit.

As described in detail in [2,6,7,21], the success of the guidance task in platooning applications strongly depends on the relationship between control, communication and sensorial systems. Regarding control and communication solutions for platooning guidance in hard non-linear trajectories different works have been carried out by the authors in the context of the COVE project [22]. The global control architecture [2] for each follower of the convoy includes a three level controller, as shown in Figure 3.

The low level is based on a set of PID controllers that regulate the speed of each active wheel. The middle level includes a servo-controller in order to ensure reliable angular and linear speeds (Vo) of the robot. The robotic units are provided with optical encoders of 500 pulses per revolution linked to each active wheel, due to its 19 cm diameter the movement resolution is 1.2 mm. In this way, odometry permits closing both the low and middle control loops. Additionally, a discrete Kalman Filter (KF estimator) is included in order to filter the noise related to measurements provided by encoders and obtaining the filtered velocity vector (VE) for control purposes. The high level generates the inputs (UHL) for the middle level, such that each robot follows the previous one warranting a security distance, and approaches the discretized poses selected by the leader in its way. The follower pose, needed to accomplish the high control level objectives, is estimated by the discrete EKF combining data coming from the odometric system and the added laser scanner.

The paper is organized into five sections: after this introduction, the sensory sources involved in the fusion process are presented in Section 2, and the discrete EKF application to the data fusion data is described in Section 3. Experimental results obtained with a real robotic convoy demonstrator are shown in Section 4, and conclusions are revealed in Section 5. The mathematical component and algorithm description are included in an appendix at the end of the paper.

## 2. Pose Estimation of Follower Units based on Odometry and Laser Combination

The convoy consists of a group of robotic units that are only equipped with relative positioning sensors (*i.e*., odometry and laser sensors). One of the units is designated as the leader unit and it is assumed that it knows its global position in the environment. Besides, all the units are nodes of a wireless local area network (Wi-Fi link) [2].

As already mentioned, global stability is achieved if each follower knows both its motion state and at least the one of the precedent unit. In order to accomplish this specification, as a first approximation, angular and linear speeds of each robot in the platoon can be estimated with a dead-reckoning process, using the encoders attached to active wheels already built on each robot. This estimation can be combined between each pair of robots, and sent through the wireless link, to obtain each unit’s relative distance and orientation to the precedent one. However, this first approach produces important drifts in the pose estimation, due to the accumulative error inherent to the dead-reckoning process, mainly in non-linear trajectories. A complementary sensorial system is therefore needed in order to better estimate the individual pose and to guarantee the reliability and stability in the guidance task performed in the convoy.

In this paper, authors propose to combine the odometric information with the laser sensor pose estimation. The laser sensor gives the relative pose (distance and orientation) from each robot to the preceding one (see Figures 1 and 2), thus avoiding cumulative errors in this information. Nevertheless, experimental results demonstrate that the uncertainty related to the pose information, calculated from the laser sensor, is bigger than the one obtained from the odometric one. However, it has to be taken into account that the error concerning the laser scanner is bounded while the one related to the odometric system is cumulative. Fusion strategies are therefore needed in order to compensate limitations and to exploit the positive characteristics related to each of the two sensory systems in the guidance application.

**Figure 4.**
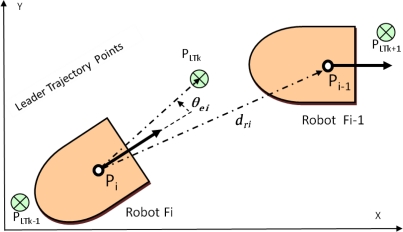
Variables involved in the high level control of the platoon: points of the leader trajectory, pose of each follower and relative position information obtained through the laser scanner.

**Figure 5.**
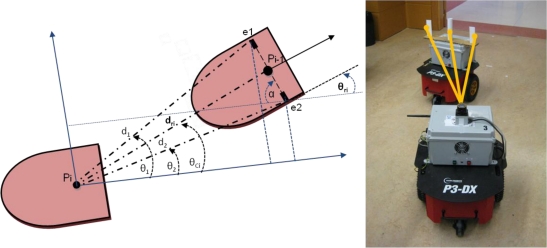
Description of the geometrical relation among the variables [*d*_1_, *θ*_1_, *d*_2_, *θ*_2_, *d_ri_*, *θ_ci_*] implied in the laser scanner relative positioning system.

The laser contributes to measure the separation distance *d_ri_* between units in the platoon, and to ascertain the correction angle *θ_ei_* needed by each unit to approach to the next pose mark *P_LTk_* sent by the leader to the rest of the convoy units [2]. These variables are illustrated in Figure 4. Complementing the laser scanner, basic artificial landmarks are placed on top of the robotic platform (see Figure 2). The landmark system includes two small planes and a cylinder between them, overhanging the compact volume of the basic platform. The cylinder is located on the dynamics reference point of the robot. It can be noticed in Figure 5 that two of the three elements included as artificial landmarks are enough to obtain both the separation distance *d_ri_* and the relative angle *θ_ri_* between robots poses *P_i_* and *P_i − 1_*.

However, working with the proposed landmark has important advantages:

- The inclusion of two planar elements minimizes the error when calculating the angle *α*, as the separation between them is big enough. The angle *α* is used to compute the relative orientation *θ_ri_* between each two consecutive units in the platoon.
- Although distance *d_ri_* can be indirectly obtained through the measures [*d*_1_, *θ*_1_] and [*d*_2_, *θ*_2_], the cylinder in the middle of the landmark eases its direct computation, improving the accuracy and computational time of the estimation.
- Thanks to its three components, the artificial landmark can be easily identified in the robot structure and from the different elements in the environment, minimizing the fault detections.

Once the artificial landmark is detected by the laser scanner on top of a follower unit, the relative distance between this unit and the one in front of it is directly obtained from the laser measures to the cylindrical structure d_ri_. From the two most external measures, detected in the landmark by the laser scanner (points *e_1_* and *e_2_* in Figure 5), the angle *α* can be calculated:

(1) $$\alpha =\text{atan}2\;\left(\frac{d_{1}\;\text{sin}\;\left(\theta_{1}\right)-d_{2}\;\text{sin}\;\left(\theta_{2}\right)}{d_{2}\;\text{cos}\;\left(\theta_{2}\right)-d_{1}\;\text{cos}\;\left(\theta_{1}\right)}\right)$$

where *atan2* is a 4-quadrant version of the inverse tangent function.

This way, the relative orientation respect to the precedent unit is obtained by the equation:

(2) $$\theta_{\text{ri}}=\frac{\pi }{2}-\alpha$$

To better understand the data fusion process, the following nomenclature should be kept in mind: *X̃* is the predicted pose based on odometry, *Z* is the estimated pose through laser measurements and *X̂* represents the corrected pose through the EKF algorithm.

Thanks to the wireless link between the units, the corrected pose *X̂_i_*_−1,_*_k_*_−1_ = [*X̂_i_*_−1,_*_k_*_−1_ *ŷ_i_*_−1,_*_k_*_−1_ *θ̂_i_*_−1,_*_k_*_−1_]*^T^* of the *F_i_*_−1_ unit at the *k-1-th* instant, is known by the unit *F_i_* at the k-th instant. In this way, using the relative laser measures, the estimated pose *Z_i_*_,_*_k_* = [*x̄_i_*_,_*_k_* *ȳ_i_*_,_*_k_* *θ̄_i_*_,_*_k_*]*^T^* of *F_i_* at the k-th instant is obtained as shown in Equations (3–5):

(3) $${\bar{\theta }}_{i,k}={\hat{\theta }}_{i-1,k-1}-\theta_{\text{ri},k}$$

(4) $${\bar{x}}_{i,k}={\hat{x}}_{i-1,k-1}-d_{\text{ri},k}\text{cos}\left({\bar{\theta }}_{i,k}{+\theta }_{\text{ci},k}\right)$$

(5) $${\bar{y}}_{i,k}={\hat{y}}_{i-1,k-1}-d_{\text{ri},k}\text{sin}\left({\bar{\theta }}_{i,k}{+\theta }_{\text{ci},k}\right)$$

**Figure 6.**
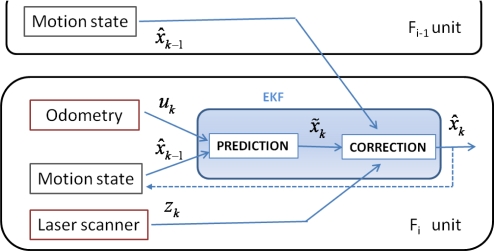
Processes and variables implied in the EKF to obtain the Fi movement state. *X̃* is the predicted pose (odometry system), *Z* is the estimated pose (laser measurements), and *X̂* represents the corrected pose (EKF algorithm).

Finally, the EKF algorithm allows one to fuse the odometric information *X̃_i_*_,_*_k_* with the laser one *Z_i_*_,_*_k_* to achieve the corrected pose *X̂_i_*_,_*_k_* of the *F_i_* unit, see Figure 6.

## 3. Discrete EKF Application to the Odometry and Laser Fusion

The best pose estimation of each follower in the convoy is achieved through a discrete Extended Kalman Filter [23–25], fusing odometry and laser scanner information. The EKF allows one to highlight the strengths of the two sensory systems. Thus, the filter develops the functions shown in Figure 6 in two steps:

- Prediction of the robot pose *X̃_k_*. The odometry information is included as input vector *U_k_* according to the speed of the active wheels at each sample time. The corrected state *X̂_k_*_−1_ in the previous sample time is also required.
- Correction of the pose estimation *X̂_k_*. This step requires the estimated pose *Z_k_* obtained once the laser scanner information is achieved.

**Figure 7.**
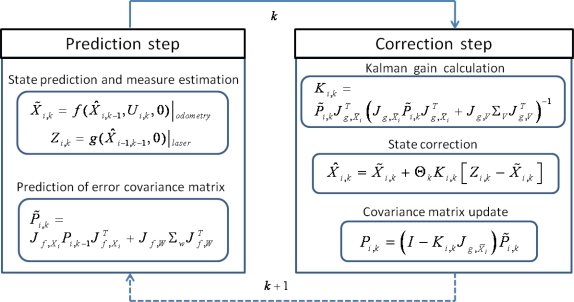
Block diagram of the implemented fusion algorithm, based on the standard EKF.

At the end of this paper, the Appendix mathematically details the specific adaptation of the discrete EKF to the problem tackled in this work, which is summarized in Figure 7.

The implemented EKF has the standard structure of this filter, except for the factor *Θ_k_*. This factor indicates the availability of the laser scanner measure: if its measures are available in a specific time *k* then *Θ_k_* = 1; otherwise *Θ_k_* = 0, and the correction step will not be executed that time *k*. The use of factor *Θ_k_* allows having timing independence for prediction and correction process [26]. In this work a sampling time of Ts = 0.05 s is constantly used for the prediction step, meanwhile the time correction will vary according to the availability of the laser scanner measures, as explained.

The different tasks developed by the filter at the EKF prediction step are summarized in the following paragraphs:

(p.1) Prediction of state *X̃_i,k_* (position and orientation) for the follower unit in an absolute positioning reference system. Dead-reckoning model based on the odometric system (f function in Figure 7), and the corrected state at previous time step *X̂*_*i*,*k*−1_, are required to obtain this predicted state.
(p.2) Estimation of measure *Z*_*i*,*k*_ from the corrected pose of the precedent unit *X̂*_*i*−1,*k*−1_ and the measurement model based on the laser scanner (*g* function in Figure 7).
(p.3) Prediction of the estimation error covariance matrix *P̃*_*i,k*_, using the corrected value of this matrix for the previous time step *P*_*i*,*k*−1_, as well as the noise covariance matrix *Σ_W_* of the odometric measurements’ model, and the two jacobians *J_f,X_* and *J_f,W_* (see the Appendix).

On the other hand, tasks developed by the discrete filter at the correction step, are the following:

(c.1) Updating the Kalman gain K_i,k_. In order to obtain this gain, some matrices have to be previously computed: estimation of the matrix P̃_i,k_, the noise covariance matrix Σ_V_ of the laser scanner measurements’ model, and the two jacobians J_g,X̄_ and J_g.V_ (see the Appendix).
(c.2) Correction of the pose state predicted value X̂_i,k_, only if the laser scanner updated measures are available, and thus Θ_k_ = 1. As it can be noticed in Figure 7, this correction is obtained weighting the difference between the position information obtained with the laser scan Z_k_, and its prediction X̃_k_ through odometry, with the Kalman gain.
(c.3) Updating the estimation error covariance matrix P_i,k_.

Among the contributions of this paper the standard discrete EKF adaptation for the pose estimation of robots in platooning guidance should be considered. Specifically the authors have developed:

- Characterization of *f* and *g* functions. The *f* one is related to the dead-reckoning model used to obtain the position information with the odometric system. The *g* function concerns the positioning system based on the relative measures of the laser scanner and the pose of the precedent unit.
- Computation of the covariance matrices that model the noise related to both sensory systems: the one related to the odometric system *Σ_W_* and the other deals with the laser scanner *Σ_V_*. In order to find these values, the angular speed of the active wheels as well as the distance and angles measured with the laser scanner has been registered in 50 experiments. The standard deviation of the related noise variables and the complete covariance matrices are computed from those registers.
- Calculation of the needed jacobians. The jacobians depending on odometry measurements: *J_f,X_* and *J_f,W_*; and the ones regarding the laser measurements: *J_g,Z_* and *J_g,V_*.

## 4. Experimental Results

In the experimental tests developed to validate the described proposal, three robotic units adapted from the original P3-DX platform (see Figures 1, 2 and 5) have been deployed. All of them are initially synchronized and currently linked by means a Wi-Fi LAN in compliance with IEEE802.11b/.11g standards [20]. The solutions carried out to mitigate the packet dropout effect were tackled by the research group in other work [27] and have also been implemented in these tests. The hardest time constraints are imposed by: the scan time of the Hokuyo device (100 ms) [16,17] and the velocity of the robot (limited to 1 m/s) [2,18].

Two types of tests are included in this section. The first one is dedicated to show the advantages of the implemented fusion technique, comparing the positioning results independently obtained with each of the two sensory systems under study. The second type focuses on the global results of control and sensorial fusion integration applied to a convoy of robots.

**Figure 8.**
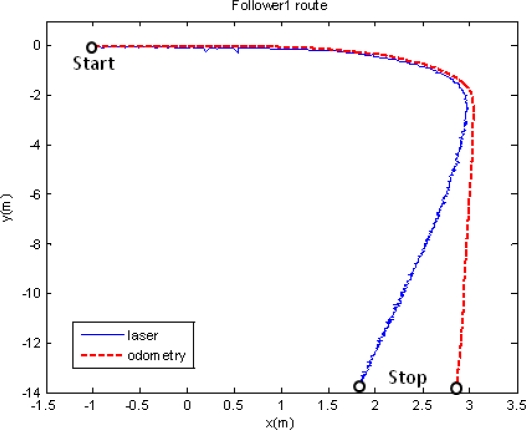
Movement developed by a P3-DX robotic unit following a leader. The red trace agrees with the odometric information of the robot. However, the laser scanner gives the more realistic blue path.

**Figure 9.**
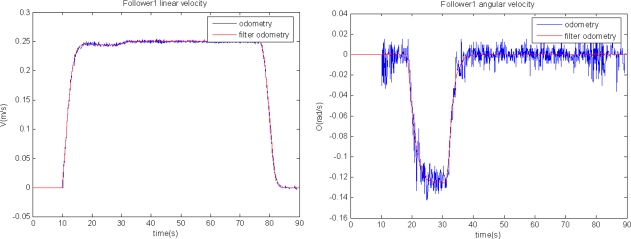
Linear and angular speed of the follower unit in the trajectory shown in Figure 8. The values registered by the odometry system are plotted in blue, and the filtered ones (used for control tasks) are in red.

In the first set of results only two units are used: the leader, programmed to track a trajectory including straight and curve segments; and a single follower. Figure 8 shows the follower path according to the different sensorial sources but without fusion application: in red it is shown the movement registered by the odometric sensory system; and in blue the one registered by the laser scanner through the relative measure respect to the leader movement. The follower unit starts at point [x = −1, y = 0]. Both depicted trajectories are close along its first straight part. Nevertheless, the information given by the two sensorial systems diverges from the moment the trajectory presents a curve path. Figure 9 shows the linear and angular speed registered by the odometric system of the follower unit tested in this first experiment. This figure allows one to demonstrate, in other way, the effect of the filter included to remove the odometry noise. In fact, it can be noticed that this noise is more relevant in the angular speed case, confirming the need of inserting the KF estimator in the global control solution (see Figure 3) for non-linear trajectory tracking.

**Figure 10.**
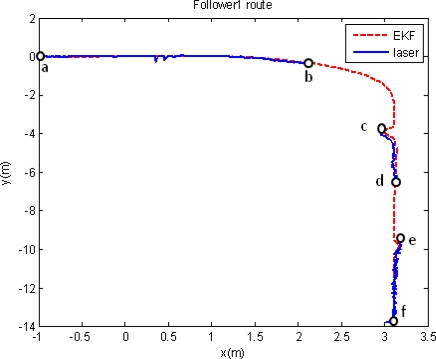
Path pursuit by the follower unit using the discrete EKF fusion proposal as part of the high level control. The blue plot shows the position information registered by the laser scanner, and the red one the location estimated by the EKF. The laser scanner is only available in intervals “*ab*”, “*cd*” and “*ef*”.

A new experiment is carried out with the same robot formation and with the same path reference. This time, the output of the fusion algorithm is applied to the high level control in the guiding architecture shown in Figure 3. In order to evaluate the discrete EKF functionality when the fusion task cannot be strictly performed, because of the lack of laser scanner measures, this sensor is blinded in some time intervals. An obstacle is inserted just in front of the scanner in some specific moments along the path. It can be then analyzed how the guidance application does correct the robot path from the drift typically generated by the stand alone use of odometry once the obstacle disappears. Figure 10 shows the path pursuit by the follower unit, using the global fusion algorithm here proposed (red line ○); the laser scanner measures are also plotted in blue, when available. In this path, along segments “*ab*”, “*cd*” and “*ef*” both sensory systems generate valid pose measures, and therefore, the fusion process is correctly developed. Besides, along segments “*bc*” and “*de*”, position information is not available in the laser scanner system, so only the prediction step of the EKF is working just using odometric information. The result of this information lack of the laser scanner is that the movement of the robot unit presents a relevant drift from its expected path when using only odometry, mainly in curve intervals. In any case, once the laser scanner measures are again available for the fusion algorithm, the guiding process is quickly adjusted to the correct path.

**Figure 11.**
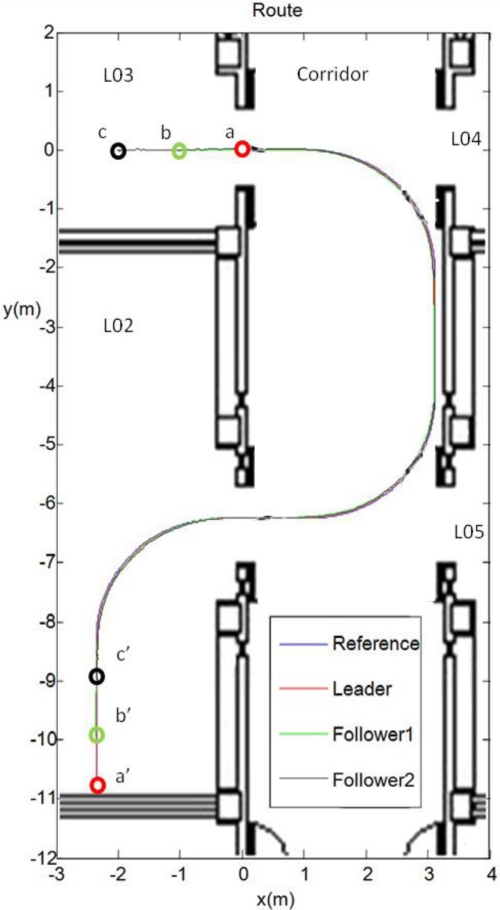
Trajectory followed by the platoon. The path described by the leader is plotted in red, the one described by the first follower is shown in green, and the one described by the second follower is plotted in black. The reference trajectory input to the leader is also shown in blue.

The second type of test is developed with a convoy of three units, as shown in Figure 2, in a more complex scenario. A video showing the overall experiment can be seen in [22]. The platoon guidance strategy, based on the three control levels and the sensorial fusion algorithm described in this paper, is implemented in the two follower units. As it can be appreciated in Figure 11, the platoon starts at L03 laboratory (where “*a*”, “*b*” and “*c*” are respectively the initial localization of each unit) and goes through a corridor to finally get into L02 laboratory (where “*a*’ ”, “*b*’ ” and “*c*’ ” are respectively the final localization of each unit). The total path followed by each robot in this platooning guidance example is depicted with different colours. The robots’ location is obtained from each “EKF sensor fusion” block (see Figure 3). It can be stated that the two follower units track with negligible error the trajectory described by the leader.

## 5. Conclusions

This work details how the proper combination of odometry and a low cost laser scanner provides the required accuracy and non-cumulative errors for indoor applications of a convoy of robotic units. First, it has been demonstrated that information coming from the proposed single sensors is not enough to accomplish the correct positioning of one or more units in cooperative guidance. In this context, the proposal presented in the paper calls for fusing odometric data (typical positioning system of a robot) with a laser scanner (added to the robotic platform together with a basic landmark structure) to achieve the guiding task of a convoy of P3-DX robots.

The contribution of the implemented discrete EKF is twofold. On one hand the inherent accumulative error due to dead-reckoning positioning is corrected by the laser measurements. On the other hand, the highest uncertainty related to the used low cost laser scanner is compensated by the lowest one of the P3-DX encoders.

As it has been demonstrated with the indoor experiments results, the sensorial fusion process is essential to maintain a safe distance between followers and to track the leader’s trajectory. The implemented solution allows one to achieve these objectives, even in situations where partial sensory information is lacking.

In summary, the paper details quantitatively how the performance of independent sensorial sources can be highly improved by means of a proper fusion algorithm, taking advantage of their best characteristics and minimizing their inherent limitations.

## Appendix

### Mathematical Description of the Implemented Discrete EKF Algorithm

The kinematical relation between the robot pose and the speed data of active wheels is not linear in a differential drive robot. Thus, in order to represent this relation in the state space, the transition and output equations respectively have to be expressed as follows:

(A.1) $$X_{i,k}=f\left(X_{i,k-1},U_{i,k},W_{k}\right)$$

(A.2) $$Z_{i,k}=g\left(X_{i-1,k-1},V_{k}\right)$$

where *X_i,k_* ∈ 𝕉^3^, is the state vector representing the absolute pose of the follower unit, with its three components *(x, y, θ)*; *X*_*i*−1,*k*_ ∈ 𝕉^3^, is the state vector of the precedent unit; *U_i,k_* ∈ 𝕉^2^, is the input vector, with its two components: angular speed *(ω_R_, ω_L_)* of the two active wheels in the platform (right and left); *Z_i,k_* ∈ 𝕉^3^, is the pose estimation through the laser scanner measures (distance and angle); *W_k_* ∈ 𝕉^2^, is the state noise vector, therefore related to the odometric system; and *V_k_* ∈ 𝕉^6^, is the measurement noise vector related to the laser scanner perception system.

As defined in the previous paragraphs, nonlinear and stochastic functions *f* and *g* are respectively related to the odometric system intrinsic to the robot, and to the laser scanner sensorial system.

As explained in Section 3, and depicted in Figure 7, two steps are periodically repeated in order to develop the EKF sensorial fusion. In the prediction step, Equations (A.3)–(A.5) are determined. A null value is supposed in this step for all noise components:

(A.3) $${\tilde{X}}_{i,k}=f\left({\hat{X}}_{i,k-1},U_{i,k},W_{k}\right)$$

(A.4) $$Z_{i,k}=g\left({\hat{X}}_{i-1,k-1},0\right)$$

(A.5) $${\tilde{P}}_{i,k}=J_{f,Xi}P_{i,k-1}J_{f,Xi}^{T}+J_{f,W}\Sigma_{w}J_{f,W}^{T}$$

In the case under study, function *f* can be defined analyzing separately the three components of *X̃_i,k_*, and thus, obtaining expressions (A.6) to (A.8) to substitute (A.3) as follows:

(A.6) $${\tilde{x}}_{i,k}={\hat{x}}_{i,k-1}+\frac{\left(\omega_{L,k}+\omega_{R,k}\right)r}{2}\cdot \mathrm{Ts}\cdot \cos\left[{\hat{\theta }}_{i,k-1}+\frac{\left(\omega_{R,k}-\omega_{L,k}\right)r}{D}\cdot \mathrm{Ts}\right]$$

(A.7) $${\tilde{y}}_{i,k}={\hat{y}}_{i,k-1}+\frac{\left(\omega_{L,k}+\omega_{R,k}\right)r}{2}\cdot \mathrm{Ts}\cdot \sin\left[{\hat{\theta }}_{i,k-1}+\frac{\left(\omega_{R,k}-\omega_{L,k}\right)r}{D}\cdot \mathrm{Ts}\right]$$

(A.8) $${\tilde{\theta }}_{i,k}={\hat{\theta }}_{i,k-1}+\frac{\left(\omega_{R,k}-\omega_{L,k}\right)r}{D}\cdot \mathrm{Ts}$$

where *Ts* is the sample time of the discrete fusion process, *r* is the active wheels radius and *D* is the distance between them. In this specific application *Ts* = 0.05 s, *r* = 0.09 m and *D* = 0.33 m.

To obtain *P̃_i,k_*, the components *J_f,Xi_*, *J_f,W_* and *Σ_W_* are needed. Expressions (A.9) and (A.12) define the two first jacobians in the context of interest:

(A.9) $$J_{f,\mathrm{Xi}}=\left[\begin{array}{ccc} \frac{\partial {\tilde{x}}_{i,k}}{\partial {\hat{x}}_{i,k-1}} & \frac{\partial {\tilde{x}}_{i,k}}{\partial {\hat{y}}_{i,k-1}} & \frac{\partial {\tilde{x}}_{i,k}}{\partial {\hat{\theta }}_{i,k-1}}\\ \frac{\partial {\tilde{y}}_{i,k}}{\partial {\hat{x}}_{i,k-1}} & \frac{\partial {\tilde{y}}_{i,k}}{\partial {\hat{y}}_{i,k-1}} & \frac{\partial {\tilde{y}}_{i,k}}{\partial {\hat{\theta }}_{i,k-1}}\\ \frac{\partial {\tilde{\theta }}_{i,k}}{\partial {\hat{x}}_{i,k-1}} & \frac{\partial {\tilde{\theta }}_{i,k}}{\partial {\hat{y}}_{i,k-1}} & \frac{\partial {\tilde{\theta }}_{i,k}}{\partial {\hat{\theta }}_{i,k-1}} \end{array}\right]=\left[\begin{array}{ccc} 1 & 0 & \frac{\partial {\tilde{x}}_{i,k}}{\partial {\hat{\theta }}_{i,k-1}}\\ 0 & 1 & \frac{\partial {\tilde{y}}_{i,k}}{\partial {\hat{\theta }}_{i,k-1}}\\ 0 & 0 & 1 \end{array}\right]$$

where:

(A.10) $$\frac{\partial {\tilde{x}}_{i,k}}{\partial {\hat{\theta }}_{i,k-1}}=-\frac{\left(\omega_{L,k}+\omega_{R,k}\right)r}{2}\cdot \mathrm{Ts}\cdot \sin\left({\hat{\theta }}_{i,k-1}+\frac{\left(\omega_{L,k}+\omega_{R,k}\right)r}{D}\cdot \mathrm{Ts}\right)$$

(A.11) $$\frac{\partial {\tilde{y}}_{i,k}}{\partial {\hat{\theta }}_{i,k-1}}=\frac{\left(\omega_{L,k}+\omega_{R,k}\right)r}{2}\cdot \mathrm{Ts}\cdot \cos\left({\hat{\theta }}_{i,k-1}+\frac{\left(\omega_{L,k}+\omega_{R,k}\right)r}{D}\cdot \mathrm{Ts}\right)$$

and:

(A.12) $$J_{f,W}=\left[\begin{array}{cc} \frac{\partial {\tilde{x}}_{i,k}}{\partial \omega_{R,k}} & \frac{\partial {\tilde{x}}_{i,k}}{\partial \omega_{L,k}}\\ \frac{\partial {\tilde{y}}_{i,k}}{\partial \omega_{R,k}} & \frac{\partial {\tilde{y}}_{i,k}}{\partial \omega_{L,k}}\\ \frac{\partial {\tilde{\theta }}_{i,k}}{\partial \omega_{R,k}} & \frac{\partial {\tilde{\theta }}_{i,k}}{\partial \omega_{L,k}} \end{array}\right]$$

where the different elements of jacobian *J_f,W_* are obtained as follows applying the kinematics relations described in (A.6) to (A.8):

(A.13) $$\begin{array}{l} \frac{\partial {\tilde{x}}_{i,k}}{\partial \omega_{R,k}}=\frac{r}{2}\cdot \mathrm{Ts}\cdot \cos\left[{\hat{\theta }}_{i,k-1}+\frac{\left(\omega_{R,k}-\omega_{L,k}\right)r}{D}\cdot \mathrm{Ts}\right]-\\ \;\;\;\;\;\;\frac{\left(\omega_{R,k}+\omega_{L,k}\right)r^{2}}{2D}\cdot {\mathrm{Ts}}^{2}\cdot \sin\left[{\hat{\theta }}_{i,k-1}+\frac{\left(\omega_{R,k}-\omega_{L,k}\right)r}{D}\cdot \mathrm{Ts}\right] \end{array}$$

(A.14) $$\begin{array}{l} \frac{\partial {\tilde{x}}_{i,k}}{\partial \omega_{L,k}}=\frac{r}{2}\cdot \mathrm{Ts}\cdot \cos\left[{\hat{\theta }}_{i,k-1}+\frac{\left(\omega_{R,k}-\omega_{L,k}\right)r}{D}\cdot \mathrm{Ts}\right]+\\ \;\;\;\;\;\;\frac{\left(\omega_{R,k}+\omega_{L,k}\right)r^{2}}{2D}\cdot {\mathrm{Ts}}^{2}\cdot \sin\left[{\hat{\theta }}_{i,k-1}+\frac{\left(\omega_{R,k}-\omega_{L,k}\right)r}{D}\cdot \mathrm{Ts}\right] \end{array}$$

(A.15) $$\begin{array}{l} \frac{\partial {\tilde{y}}_{i,k}}{\partial \omega_{R,k}}=\frac{r}{2}\cdot \mathrm{Ts}\cdot \sin\left[{\hat{\theta }}_{i,k-1}+\frac{\left(\omega_{R,k}-\omega_{L,k}\right)r}{D}\cdot \mathrm{Ts}\right]+\\ \;\;\;\;\;\;\frac{\left(\omega_{R,k}+\omega_{L,k}\right)r^{2}}{2D}\cdot {\mathrm{Ts}}^{2}\cdot \cos\left[{\hat{\theta }}_{i,k-1}+\frac{\left(\omega_{R,k}-\omega_{L,k}\right)r}{D}\cdot \mathrm{Ts}\right] \end{array}$$

(A.16) $$\begin{array}{l} \frac{\partial {\tilde{y}}_{i,k}}{\partial \omega_{L,k}}=\frac{r}{2}\cdot \mathrm{Ts}\cdot \sin\left[{\hat{\theta }}_{i,k-1}+\frac{\left(\omega_{R,k}-\omega_{L,k}\right)r}{D}\cdot \mathrm{Ts}\right]-\\ \;\;\;\;\;\;\frac{\left(\omega_{R,k}+\omega_{L,k}\right)r^{2}}{2D}\cdot {\mathrm{Ts}}^{2}\cdot \cos\left[{\hat{\theta }}_{i,k-1}+\frac{\left(\omega_{R,k}-\omega_{L,k}\right)r}{D}\cdot \mathrm{Ts}\right] \end{array}$$

(A.17) $$\frac{\partial {\tilde{\theta }}_{i,k}}{\partial \omega_{R,k}}=\frac{r}{D}\cdot \mathrm{Ts}$$

(A.18) $$\frac{\partial {\tilde{\theta }}_{i,k}}{\partial \omega_{L,k}}=-\frac{r}{D}\cdot \mathrm{Ts}$$

Besides, the noise covariance matrix related to the odometric information *Σ_W_* is empirically and statistically defined (see Section III), resulting in this application as follows:

(A.19) $$\Sigma_{w}=\left[\begin{array}{cc} \Sigma_{\mathrm{enc}} & 0\\ 0 & \Sigma_{\mathrm{enc}} \end{array}\right],\text{ being }\Sigma_{\text{enc}}=3.8125\;106\;{\text{rad}}^{2}/s^{2}$$

Once Equation (A.3) has been detailed, we focus on the Equation (A.4) through the g function. It includes terms of the precedent unit *F_i−1_* and laser scanner measures, as it has been described in Equations (3) to (5), and has been depicted in Figure 5. The estimation pose, which is based on the laser sensorial system, includes three components *Z_i,k_* = [*x̄_i,k_*, *ȳ_i,k_*, *θ̄_i,k_*]*^T^* that are going to be analyzed separately:

(A.20) $${\bar{x}}_{i,k}={\hat{x}}_{i-1,k-1}-d_{\mathrm{ri},k}\;\cos\;\left[\theta_{\mathrm{ci},k}+{\bar{\theta }}_{i,k-1}-\frac{\pi }{2}+\mathrm{atan}2\;\left(\frac{d_{1,k}\;\sin\;\left(\theta_{1,k}\right)-d_{2,k}\;\sin\;\left(\theta_{2,k}\right)}{d_{2,k}\;\cos\;\left(\theta_{2,k}\right)-d_{1,k}\;\cos\;\left(\theta_{1,k}\right)}\right)\right]$$

(A.21) $${\bar{y}}_{i,k}={\hat{y}}_{i-1,k-1}-d_{\mathrm{ri},k}\;\sin\;\left[\theta_{\mathrm{ci},k}+{\bar{\theta }}_{i-1,k-1}-\frac{\pi }{2}+\;\mathrm{atan}2\;\left(\frac{d_{1,k}\;\sin\;\left(\theta_{1,k}\right)-d_{2,k}\;\sin\;\left(\theta_{2,k}\right)}{d_{2,k}\;\cos\;\left(\theta_{2,k}\right)-d_{1,k}\;\cos\;\left(\theta_{1,k}\right)}\right)\right]$$

(A.22) $${\bar{\theta }}_{i,k}={\hat{\theta }}_{i-1,k-1}-\frac{\pi }{2}+\mathrm{atan}2\;\left(\frac{d_{1,k}\;\sin\;\left(\theta_{1,k}\right)-d_{2,k}\;\sin\;\left(\theta_{2,k}\right)}{d_{2,k}\;\cos\;\left(\theta_{2,k}\right)-d_{1k}\;\cos\;\left(\theta_{1,k}\right)}\right)$$

At this point, the correction step is tackled. The Kalman gain *K_i,k_*, the estimation error covariance matrix *P_i,k_* and the corrected pose *X̂_i,k_* are evaluated, as shown in Figure 7. The Kalman gain is obtained from the jacobians *J_g,X̄_* and *J_g,V_*, whose values are calculated as follows:

(A.23) $$J_{g,\bar{X}}=\left[\begin{array}{ccc} \frac{\partial {\bar{x}}_{i,k}}{\partial {\bar{x}}_{i,k}} & \frac{\partial {\bar{x}}_{i,k}}{\partial {\bar{y}}_{i,k}} & \frac{\partial {\bar{x}}_{i,k}}{\partial {\bar{\theta }}_{i,k}}\\ \frac{\partial {\bar{y}}_{i,k}}{\partial {\bar{x}}_{i,k}} & \frac{\partial {\bar{y}}_{i,k}}{\partial {\bar{y}}_{i,k}} & \frac{\partial {\bar{y}}_{i,k}}{\partial {\bar{\theta }}_{i,k}}\\ \frac{\partial {\bar{\theta }}_{i,k}}{\partial {\bar{x}}_{i,k}} & \frac{\partial {\bar{\theta }}_{i,k}}{\partial {\bar{y}}_{i,k}} & \frac{\partial {\bar{\theta }}_{i,k}}{\partial {\bar{\theta }}_{i,k}} \end{array}\right]=\left[\begin{array}{ccc} 1 & 0 & \frac{\partial {\bar{x}}_{i,k}}{\partial {\bar{\theta }}_{i,k}}\\ 0 & 1 & \frac{\partial {\bar{y}}_{i,k}}{\partial {\bar{\theta }}_{i,k}}\\ 0 & 0 & 1 \end{array}\right]$$

where:

(A.24) $$\frac{\partial {\bar{x}}_{i,k}}{\partial {\bar{\theta }}_{i,k}}=d_{\mathrm{ri},k}\;\sin\;\left(\theta_{\mathrm{ci},k}+{\bar{\theta }}_{i,k}\right)$$

(A.25) $$\frac{\partial {\bar{y}}_{i,k}}{\partial {\bar{\theta }}_{i,k}}=-d_{\mathrm{ri},k}\cos\left(\theta_{\mathrm{ci},k}+{\bar{\theta }}_{i,k}\right)$$

and:

(A.26) $$J_{g,V}=\left[\begin{array}{cccccc} \frac{\partial {\bar{x}}_{i,k}}{\partial d_{\mathrm{ri},k}} & \frac{\partial {\bar{x}}_{i,k}}{\partial \theta_{\mathrm{ci},k}} & \frac{\partial {\bar{x}}_{i,k}}{\partial d_{1,k}} & \frac{\partial {\bar{x}}_{i,k}}{\partial \theta_{1,k}} & \frac{\partial {\bar{x}}_{i,k}}{\partial d_{2,k}} & \frac{\partial {\bar{x}}_{i,k}}{\partial \theta_{2,k}}\\ \frac{\partial {\bar{y}}_{i,k}}{\partial d_{\mathrm{ri},k}} & \frac{\partial {\bar{y}}_{i,k}}{\partial \theta_{\mathrm{ci},k}} & \frac{\partial {\bar{y}}_{i,k}}{\partial d_{1,k}} & \frac{\partial {\bar{y}}_{i,k}}{\partial \theta_{1,k}} & \frac{\partial {\bar{y}}_{i,k}}{\partial d_{2,k}} & \frac{\partial {\bar{y}}_{i,k}}{\partial \theta_{2,k}}\\ \frac{\partial {\bar{\theta }}_{i,k}}{\partial d_{\mathrm{ri},k}} & \frac{\partial {\bar{\theta }}_{i,k}}{\partial \theta_{\mathrm{ci},k}} & \frac{\partial {\bar{\theta }}_{i,k}}{\partial d_{1,k}} & \frac{\partial {\bar{\theta }}_{i,k}}{\partial \theta_{1,k}} & \frac{\partial {\bar{\theta }}_{i,k}}{\partial d_{2,k}} & \frac{\partial {\bar{\theta }}_{i,k}}{\partial \theta_{2,k}} \end{array}\right]$$

Expressions from (A.29) to (A.46) allow one to achieve the 18 elements of *J_g,V_*, knowing that:

(A.27) $$a=d_{1,k}\;\sin\;\left(\theta_{1,k}\right)-d_{2,k}\;\sin\;\left(\theta_{2,k}\right)$$

(A.28) $$b=d_{2,k}\;\cos\;\left(\theta_{2,k}\right)-d_{1,k}\;\cos\;\left(\theta_{1,k}\right)$$

Then:

(A.29) $$\frac{\partial {\bar{x}}_{i,k}}{\partial d_{\mathrm{ri},k}}=-\;\text{cos}\;\left[\theta_{\mathrm{ci},k}+{\hat{\theta }}_{i-1,k-1}-\frac{\pi }{2}+\mathrm{atan}2\;\left(\frac{d_{1,k}\;\text{sin}\;\left(\theta_{1,k}\right)-d_{2,k}\;\text{sin}\;\left(\theta_{2,k}\right)}{d_{2,k}\;\text{cos}\;\left(\theta_{2,k}\right)-d_{1,k}\;\text{cos}\;\left(\theta_{1,k}\right)}\right)\right]$$

(A.30) $$\frac{\partial {\bar{x}}_{i,k}}{\partial \theta_{\mathrm{ci},k}}=d_{\mathrm{ri}}\;\text{sin}\;\left[\theta_{\mathrm{ci},k}+{\hat{\theta }}_{i-1,k-1}-\frac{\pi }{2}+\mathrm{atan}2\;\left(\frac{d_{1,k}\;\text{sin}\;\left(\theta_{1,k}\right)-d_{2,k}\;\text{sin}\;\left(\theta_{2,k}\right)}{d_{2,k}\;\text{cos}\;\left(\theta_{2,k}\right)-d_{1,k}\;\text{cos}\;\left(\theta_{1,k}\right)}\right)\right]$$

(A.31) $$\frac{\partial {\bar{x}}_{i,k}}{\partial d_{1,k}}=d_{\mathrm{ri}}\;\text{sin}\;\left[\theta_{\mathrm{ci},k}+{\hat{\theta }}_{i-1,k-1}-\frac{\pi }{2}+\mathrm{atan}2\;\left(\frac{a}{b}\right)\right]\frac{\;\text{sin}\;\left(\theta_{1,k}\right)\;b+\;\text{cos}\;\left(\theta_{1,k}\right)a}{a^{2}+b^{2}}$$

(A.32) $$\frac{\partial {\bar{x}}_{i,k}}{\partial \theta_{1,k}}=d_{\mathrm{ri}}\;\text{sin}\;\left[\theta_{\mathrm{ci},k}+{\hat{\theta }}_{i-1,k-1}-\frac{\pi }{2}+\mathrm{atan}2\;\left(\frac{a}{b}\right)\right]\frac{\;\text{cos}\;\left(\theta_{1,k}\right)\;b-\;\text{sin}\;\left(\theta_{1,k}\right)a}{a^{2}+b^{2}}d_{1,k}$$

(A.33) $$\frac{\partial {\bar{x}}_{i,k}}{\partial d_{2,k}}=d_{\mathrm{ri}}\;\text{sin}\;\left[\theta_{\mathrm{ci},k}+{\hat{\theta }}_{i-1,k-1}-\frac{\pi }{2}+\mathrm{atan}2\;\left(\frac{a}{b}\right)\right]\frac{-\;\text{sin}\;\left(\theta_{2,k}\right)\;b-\;\text{cos}\;\left(\theta_{2,k}\right)a}{a^{2}+b^{2}}$$

(A.34) $$\frac{\partial {\bar{x}}_{i,k}}{\partial \theta_{2,k}}=d_{\mathrm{ri}}\;\text{sin}\;\left[\theta_{\mathrm{ci},k}+{\hat{\theta }}_{i-1,k-1}-\frac{\pi }{2}+\mathrm{atan}2\;\left(\frac{a}{b}\right)\right]\frac{\;\text{sin}\;\left(\theta_{2,k}\right)a-\;\text{cos}\;\left(\theta_{2,k}\right)b}{a^{2}+b^{2}}d_{2,k}$$

(A.35) $$\frac{\partial {\bar{y}}_{i,k}}{\partial d_{\mathrm{ri},k}}=-\;\text{sin}\;\left[\theta_{\mathrm{ci},k}+{\hat{\theta }}_{i-1,k-1}-\frac{\pi }{2}+\mathrm{atan}2\;\left(\frac{d_{1,k}\;\text{sin}\;\left(\theta_{1,k}\right)-d_{2,k}\;\text{sin}\;\left(\theta_{2,k}\right)}{d_{2,k}\;\text{cos}\;\left(\theta_{2,k}\right)-d_{1,k}\;\text{cos}\;\left(\theta_{1,k}\right)}\right)\right]$$

(A.36) $$\frac{\partial {\bar{y}}_{i,k}}{\partial \theta_{\mathrm{ci},k}}=-d_{\mathrm{ri}}\;\text{cos}\;\left[\theta_{\mathrm{ci},k}+{\hat{\theta }}_{i-1,k-1}-\frac{\pi }{2}+\mathrm{atn}2\;\left(\frac{d_{1,k}\;\text{sin}\;\left(\theta_{1,k}\right)-d_{2,k}\;\text{sin}\;\left(\theta_{2,k}\right)}{d_{2,k}\;\text{cos}\;\left(\theta_{2,k}\right)-d_{1,k}\;\text{cos}\;\left(\theta_{1,k}\right)}\right)\right]$$

(A.37) $$\frac{\partial {\bar{y}}_{i,k}}{\partial d_{1,k}}=-d_{\mathrm{ri}}\;\text{cos}\;\left[\theta_{\mathrm{ci},k}+{\hat{\theta }}_{i-1,k-1}-\frac{\pi }{2}+\mathrm{atan}2\;\left(\frac{a}{b}\right)\right]\frac{\;\text{sin}\;\left(\theta_{1,k}\right)\;b+\;\text{cos}\;\left(\theta_{1,k}\right)a}{a^{2}+b^{2}}$$

(A.38) $$\frac{\partial {\bar{y}}_{i,k}}{\partial \theta_{1,k}}=-d_{\mathrm{ri}}\;\text{cos}\;\left[\theta_{\mathrm{ci},k}+{\hat{\theta }}_{i-1,k-1}-\frac{\pi }{2}+\mathrm{atn}2\;\left(\frac{a}{b}\right)\right]\frac{\;\text{cos}\;\left(\theta_{1,k}\right)\;b-\;\text{sin}\;\left(\theta_{1,k}\right)a}{a^{2}+b^{2}}d_{1,k}$$

(A.39) $$\frac{\partial {\bar{y}}_{i,k}}{\partial d_{2,k}}=-d_{\mathrm{ri}}\;\text{cos}\;\left[\theta_{\mathrm{ci},k}+{\hat{\theta }}_{i-1,k-1}-\frac{\pi }{2}+\mathrm{atan}2\;\left(\frac{a}{b}\right)\right]\frac{-\;\text{sin}\;\left(\theta_{2,k}\right)\;b-\;\text{cos}\;\left(\theta_{2,k}\right)a}{a^{2}+b^{2}}$$

(A.40) $$\frac{\partial {\bar{y}}_{i,k}}{\partial \theta_{2,k}}=-d_{\mathrm{ri}}\;\text{cos}\;\left[\theta_{\mathrm{ci},k}+{\hat{\theta }}_{i-1,k-1}-\frac{\pi }{2}+\mathrm{atn}2\;\left(\frac{a}{b}\right)\right]\frac{\;\text{sin}\;\left(\theta_{2,k}\right)\;a-\;\text{cos}\;\left(\theta_{2,k}\right)\;b}{a^{2}+b^{2}}d_{2,k}$$

(A.41) $$\frac{\partial {\bar{\theta }}_{i,k}}{\partial d_{c,k}}=0$$

(A.42) $$\frac{\partial {\bar{\theta }}_{i,k}}{\partial \theta_{c,k}}=0$$

(A.43) $$\frac{\partial {\bar{\theta }}_{i,k}}{\partial d_{1,k}}==\frac{\sin\;\left(\theta_{1,k}\right)\;b+\cos\;\left(\theta_{1,k}\right)a}{a^{2}+b^{2}}$$

(A.44) $$\frac{\partial {\bar{\theta }}_{i,k}}{\partial \theta_{1,k}}=\frac{\cos\;\left(\theta_{1,k}\right)\;b-\mathrm{sen}\;\left(\theta_{1,k}\right)a}{a^{2}+b^{2}}d_{1,k}$$

(A.45) $$\frac{\partial {\bar{\theta }}_{i,k}}{\partial d_{2,k}}=\frac{-\sin\;\left(\theta_{2,k}\right)\;b-\cos\;\left(\theta_{2,k}\right)a}{a^{2}+b^{2}}$$

(A.46) $$\frac{\partial {\bar{\theta }}_{i,k}}{\partial \theta_{2,k}}=\frac{-\cos\;\left(\theta_{2,k}\right)\;b+\mathrm{sen}\;\left(\theta_{2,k}\right)a}{a^{2}+b^{2}}d_{2,k}$$

The noise covariance matrix regarding the laser scanner information *Σ_V_* is statistically and empirically defined through angular and distance parameters as described in Section III. In the case under study, the results are:

(A.47) $$\Sigma_{V}=\left[\begin{array}{cccccc} \Sigma_{d} & 0 & 0 & 0 & 0 & 0\\ 0 & \Sigma_{\theta } & 0 & 0 & 0 & 0\\ 0 & 0 & \Sigma_{d} & 0 & 0 & 0\\ 0 & 0 & 0 & \Sigma_{\theta } & 0 & 0\\ 0 & 0 & 0 & 0 & \Sigma_{d} & 0\\ 0 & 0 & 0 & 0 & 0 & \Sigma_{\theta } \end{array}\right]$$

where *Σ_d_* = 8.286 · 10^−6^ m^2^, and *Σ_θ_* = 7.615 · 10^−5^ rad^2^.

All data generated as described in previous paragraphs are needed to correct *F_i_* pose, through expression (A.48). This equation performs the correction of state vector *X̂_i,k_*, from its prediction *X̃_i,k_* based on the odometric information and the pose deduced from the laser scanner measures *Z_i,k_*:

(A.48) $${\hat{X}}_{i,k}={\tilde{X}}_{i,k}+\Theta_{k}K_{i,k}\left[Z_{i,k}-{\tilde{X}}_{i,k})\right]$$

where *Θ_k_* essential functionality is described in Section 3.

The correction step ends updating the error estimation covariance matrix, as follows:

(A.49) $$P_{i,k}=\left(I-K_{i,k}J_{g,\bar{X}}\right){\tilde{P}}_{i,k}$$

being I the identity matrix.

## References

[1] Haoyao C (2009). Towards Multi-Robot Formations: Study on Vision Based Localization Systems. http://hdl.handle.net/2031/5836.

[2] Santos C, Espinosa F, Pizarro D, Valdés F, Santiso E, Díaz I Fuzzy Decentralized Control for Guidance of a Convoy of Robots in Non-Linear Trajectories.

[3] Aulinas J, Petillot Y, Salvi J, Lladó X The SLAM Problem: A Survey.

[4] Borenstein J, Everett HR, Feng L, Wehe D (1997). Mobile robot positioning: Sensors and techniques. J. Rob. Syst.

[5] Fernandez I, Mazo M, Lazaro JL, Pizarro D, Santiso E, Martin P, Losada C (2007). Guidance of a mobile robot using an array of static cameras located in the environment. Auton Robots.

[6] Kato S, Tsugawa S, Tokuda K, Matsui T, Fujii H (2002). Vehicle control algorithms for cooperative driving with automated vehicles and intervehicle communications. IEEE Trans. Intell. Transp. Syst.

[7] Stankovic SS, Stanojevic MJ, Siljak DD (2000). Decentralized overlapping control of a platoon of vehicles. IEEE Trans. Control Syst. Technol.

[8] Huppe X, de Lafontaine J, Beauregard M, Michaud F Guidance and Control of a Platoon of Vehicles Adapted to Changing Environment Conditions.

[9] Espinosa F, Awawdeh AMH, Mazo M, Rodríguez JM, Bocos A, Manzano M Reduction of Lateral and Longitudinal Oscillations of Vehicles’ Platooning by Means of Decentralized Overlapping Control.

[10] Rodriguez M, Iglesias R, Espinosa F, Quintia P, Regueiro CV, Valdes F Learning Proposal Based on Reinforcement for Collaborative Tasks: Robot Convoy Formation.

[11] Cho YK, Youn J-H Wireless Sensor-driven Intelligent Navigation Robots for Indoor Construction Site Security and Safety.

[12] Sequeira G (2007). Vision Based Leader-Follower Formation Control for Mobile Robots. http://scholarsmine.mst.edu/thesis/pdf/Sequeira_09007dcc804429d4.pdf.

[13] Hashimoto M, Oba F, Tomiie T (1999). Mobile robot localization using color signboard. Mechatronics J.

[14] Farrington NM, Nguyen HG, Pezeshkian N (2004). Intelligent behaviors for a convoy of indoor mobile robots operating in unknown environments. Proc SPIE.

[15] Nguyen HG, Farrington N, Pezeshkian N (2004). Maintaining Communication Link for Tactical Ground Robots.

[16] Range-finder laser scanner URG-04LX. http://www.acroname.com/robotics/parts/R283-HOKUYO-LASER1.html.

[17] Vázquez-Martín R, Núñez P, Bandera A, Sandoval F (2009). Curvature-based environment description for robot navigation using laser range sensors. Sensors.

[18] MobileRobots P3-DX Units.

[19] Pizarro D, Mazo M, Santiso E, Marron M, Jimenez D, Cobreces S, Losada C (2010). Localization of mobile robots using odometry and an external vision sensor. Sensors.

[20] Espinosa F, Salazar M, Bocos A, Valdés F, Iglesias R Design and Implementation of a Communication Architecture based on Player/Stage for Telerobotics Operation of P3-DX units.

[21] Espinosa F, Salazar M, Pizarro D, Valdés F, Mollet N (2010). Electronics proposal for telerobotics operation of P3-DX units. Remote and Telerobotics.

[22] COVE Research Project.

[23] Terejanu GA (2003). Extended Kalman Filter Tutorial.

[24] Sasiadek JZ, Hartana P Sensor Data Fusion Using Kalman Filter.

[25] Kiriy E, Buehler M (2002). Three-State Extended Kalman Filter for Mobile Robot Localization.

[26] Smyth A, Wu M (2007). Multi-rate Kalman filtering for the data fusion of displacement and acceleration response measurements in dynamic system monitoring. Mech. Syst. Signal Process.

[27] Bocos A, Espinosa F, Salazar M, Valdés F Compensation of Channel Packet Dropout Based on TVKF Optimal Estimator for Robotics Teleoperation.

